# Interactions between β-Catenin and the HSlo Potassium Channel Regulates HSlo Surface Expression

**DOI:** 10.1371/journal.pone.0028264

**Published:** 2011-12-14

**Authors:** Shumin Bian, Jun-Ping Bai, Hannah Chapin, Cathy Le Moellic, Huiping Dong, Michael Caplan, Fred J. Sigworth, Dhasakumar S. Navaratnam

**Affiliations:** 1 Department of Neurology, Yale University School of Medicine, New Haven, Connecticut, United States of America; 2 Department of Cellular and Molecular Physiology, Yale University School of Medicine, New Haven, Connecticut, United States of America; Northwestern University Feinberg School of Medicine, United States of America

## Abstract

**Background:**

The large conductance calcium-activated potassium channel alpha-subunit (Slo) is widely distributed throughout the body and plays an important role in a number of diseases. Prior work has shown that Slo, through its S10 region, interacts with β-catenin, a key component of the cytoskeleton framework and the Wnt signaling pathway. However, the physiological significance of this interaction was not clear.

**Methodology/Principal Findings:**

Using a combination of proteomic and cell biology tools we show the existence of additional multiple binding sites in Slo, and explore in detail β-catenin interactions with the S10 region. We demonstrate that deletion of this region reduces Slo surface expression in HEK cells, which indicates that interaction with beta-catenin is important for Slo surface expression. This is confirmed by reduced expression of Slo in HEK cells and chicken (Gallus gallus *domesticus* leghorn white) hair cells treated with siRNA to β-catenin. HSlo reciprocally co-immunoprecipitates with β-catenin, indicating a stable binding between these two proteins, with the S10 deletion mutant having reduced binding with β-catenin. We also observed that mutations of the two putative GSK phosphorylation sites within the S10 region affect both the surface expression of Slo and the channel's voltage and calcium sensitivities. Interestingly, expression of exogenous Slo in HEK cells inhibits β-catenin-dependent canonical Wnt signaling.

**Conclusions and Significance:**

These studies identify for the first time a central role for β-catenin in mediating Slo surface expression. Additionally we show that Slo overexpression can lead to downregulation of Wnt signaling.

## Introduction

The large conductance Ca^2+^ activated potassium channel is a ubiquitous channel that plays numerous physiological roles [1] [2] [3]. Disordered channel function has been linked to diseases as diverse as hypertension, epilepsy and movement disorders. This channel is sensitive to changes in membrane voltage and intracellular Ca^2+^ concentrations [4]. It is also notable for its large single channel conductance ranging from 100–220 pS. The molecular identity of this channel was established by the cloning of the *Drosophila* homolog Slowpoke (Slo) [5]. The Slo protein consists of 6 transmembrane regions that are analogous to voltage activated potassium channels and a large intracellular C-terminus [5] [6]. The C-terminus contains the Ca^2+^ binding “bowl” together with the adjacent S10 region [5] [6]. It is now accepted that the core of this channel is formed by tetrameric association of alpha subunits encoded by this single gene [7].

The Slo protein associates with a number of ancillary subunits and other proteins that affect ion channel kinetics and subcellular localization [8]. The best studied among these subunits are the beta subunits 1–4, which affect both its kinetics and surface expression [9]. Cereblon is another protein that is important for the surface expression of Slo [10]. Other proteins that attach to Slo include Rack1 and cortactin, which mediate its interactions with protein kinase C and tyrosine kinases respectively [11] [12]. Caveolin-1 associates with Slo and may direct the channels to caveolae [13]. The ankyrin repeat protein ANKRA binds to Slo and affects its kinetics [14] . Syntaxin1 binds to Slo and decreases its voltage and apparent calcium sensitivities [15]. β-catenin was also identified as a Slo interacting protein [16].

β -catenin is a part of the cadherin cell adhesion complex and also mediates signaling by the Wnt pathway [17]. Work by Lesage et al., (2004) seeking to identify mechanisms of physically coupling Slo to voltage gated calcium channels (Ca_v_), identified beta-catenin as interacting with Slo [16]. Previous work has shown beta-catenin interactions with Lin7/Velis/MALS, whose interaction partner Lin2/CASK also binds voltage-gated Ca^2+^ channels [18] [19] [20]. Lesage et al. performed a yeast two-hybrid screen using 467 amino acids of the intracellular C-terminus of Slo as bait. Three clones were identified, all of which encoded β-catenin. The authors went on to show that this interaction was mediated by the S10 region of Slo, and by the ninth armadillo repeat and a poorly defined region of the C-terminus distal to the ninth armadillo repeat in β-catenin. While they were able to demonstrate interactions between Slo and β-catenin in vivo by pull-down assays, they were unable to demonstrate a direct interaction between these proteins by heterologous expression in COS cells [16]. Thus, these authors established that Slo and β-catenin were associated, although the physiological significance of this interaction was unclear.

We present here data that extends this work. We demonstrate that the interaction between Slo and β-catenin is important for Slo surface expression in HEK-293 cells and chick hair cells in culture. Both deletion of the proposed S10 interacting region on HSlo and siRNA knockdown of β-catenin reduces Slo surface expression in HEK cells. Similarly, siRNA mediated knockdown of β-catenin resulted in decreased Slo on the surface of chick hair cells. Mutations of two putative GSK phosphorylation sites within the S10 region alter the surface expression of Slo in HEK cells, while also affecting the biophysical properties of the expressed channels. Since β-catenin is a major component in the Wnt signaling pathway, we also explored the effect of channel expression on Wnt signaling. Interestingly, expression of exogenous HSlo inhibits canonical Wnt signaling in HEK cells. This shows that Slo may modulate the Wnt signaling pathway presumably through its binding and immobilization of β-catenin.

## Materials and Methods

### Chemicals

Iberiotoxin-LC-Biotin was a kind gift from Dr. John-Paul Bingham (University of Hawaii). N-dodecyl beta-D-maltoside (DDM) was obtained from CalBiochem.

### Antibodies

Antibody suppliers were as follows: mouse anti-Flag M2 and rabbit anti-Flag (Sigma); anti-BK_Ca_ (Alamone Labs); mouse β-catenin (BD Transduction Laboratories); mouse GSK3β (BD Transduction Laboratories); rabbit GSK3β (Cell Signaling); mouse anti-vinculin (Chemicon). bSloC was an affinity-purified rabbit antibody to the last C-terminal 21 amino acids of the bovine Slo channel.

### Cell Lines and cell culture

HEK293 cells and H28 (NCI-H28) cells were from American Type Culture Collection (ATCC). 293T was a specially maintained cell line, which is responsive to stimulation of Wnt signaling [21]. HEK293 and 293T cells were grown in DMEM medium (Gibco) supplemented with 10% fetal bovine serum and 50 U/mL penicillin/streptomycin (Gibco), in a humidified incubator at 37°C with 5% CO2. Transfections were done using Lipofectamine 2000 (Invitrogen), or Superfect (Qiagen) according to manufacturers' instructions. The stable HSloHEK293 cell line used was described previously [22] . HSlo S10 deletion mutants and point mutants were stably transfected using G418 selection. HEK293-HSlo and mutant cells had 0.6 mg/mL Geneticin (G418, from Gibco) added for selective growth. H28 cells were grown in RPMI-1640 medium (Gibco) supplemented with 10% fetal bovine serum, 50 U/mL penicillin/streptomycin (Gibco). Reverse transfection was used for transfecting HSlo/EGFP in H28 cells.

### Mutagenesis

A pcDNA3 vector encoding a His6-Flag-epitope tagged version of the human BK channel (HSlo) originally cloned by Wallner et al. (1995) was kindly supplied by Dr. Andrew Tinker, University College London [23]. The FLAG sequence was inserted at its extracellular N-terminus. The QuikChange II site-directed mutagenesis kit (Stratagene) was used to make deletion mutations and point mutations following the manufacturer's instructions. For the S10 deletion mutant the primer sequences were: forward primer 5′ctgtacctcacgcagcccaatgacaatatcctcaccctgatacgtac gctggtgacc 3′, backward primer 5′ggtcaccagcgtacgtatcagggtgaggatattgtcattgggctgc gtgaggtacag 3′. For S918A and S922A mutant the forward primer was 5′ gcatttgccgtcgctgtcctggacgctctcatgagcgctacgtacttca atg 3′, and the backward primer 5′cattgaagtacgtagcgctcatgagagcgtccaggacagcgacg gcaaatgc 3′.For S918D and S922D the forward primer was 5′ ggacagcatttgccgtcgacgtcctggacgatctcatgagcgcgacg 3′ and the backward primer 5′ cgtcgcgctcatgagatcgtccaggacgtcgacggcaaatgctgtcc 3′. The mutant inserts were sequenced in their entirety after mutagenesis. β-catenin-EGFP was subcloned from a LZRS-GFP-bCAT vector (a kind gift from Dr. Aimin Jiang, Department of Cell Biology, Yale School of Medicine) into pcDNA3.1_hygro(+) vector using BamHI/NotI restriction sites.

### Immunofluorescence detection and FACS sorting

Surface labeling of HSlo/mutant in transfected HEK293 cells with Ibtx-LC-biotin was done as described previously [22]. For surface labeling of HSlo/mutants with anti-Flag antibody in HEK293 cells were grown in a 12-well culture plate on autoclaved cover slips treated with sterile poly-D-lysine hydrobromide (Sigma). Cells were washed twice with phosphate buffered saline pH 7.4 (PBS), and incubated in PBS containing 5% fetal bovine serum (FBS) (Gibco) and 5 µg/ml anti-Flag antibody for 1 hour at room temperature. Cells were washed three times in PBS and incubated for 40 minutes at room temperature in the same buffer containing 3 µg/ml secondary antibody-AlexaFluor conjugates (Molecular Probes). The cells were washed a further three times in PBS and fixed in 3% formaldehyde in PBS for 20 minutes before mounting. To label intracellular proteins cells were fixed after surface labeling for one hour. They were then permeabilized in 0.1% Triton X100 (Calbiochem) in PBS with 5% FBS, followed by intracellular labeling with antibodies.

Slo and β-catenin staining in hair cells was done as described previously [24]. β -catenin was labeled using TRITC conjugated anti β-catenin antibody (BD). BK channels were labeled using a mouse anti-Slo antibody (BD) that was detected in turn with an Alexa 647 conjugated anti-mouse antibody (Molecular Probes). Hair cells were imaged along the z axis at the same tonotopic location (3 mm from the apical end of the basilar papilla). We used Zeiss Image Examiner software to quantify fluorescence. Slo clusters were defined by fluorescence intensity. The minimal fluorescence intensity of Slo clusters (400 A.U.) were determined by random sampling of over 100 clusters from tall hair cells of control cochlea and cochlea treated with β-catenin siRNA and used as a cutoff to identify clusters. Immunofluorescence images were taken on a Zeiss LSM 510 or LSM-Meta Laser Scanning confocal microscope, or a Nikon Eclipse TE2000-E inverted epifluorescence microscope.

For FACS analysis cells were detached from culture dishes using Ca^2+^ free PBS, and HSlo surface expression detected as described above. FACS experiments were done as previously described on a FACS Caliber machine (BD BioSciences) using FlowJo software [25].

### HSlo purification, western blotting and immunoprecipitation

HSlo was purified from a stable HSlo HEK cell membrane by affinity chromatography using an M2 anti-Flag/agarose (Sigma) column. Cells were lysed in 16 mM DDM, 250 mM KCl, protease inhibitor cocktail (Sigma). After washing with 20 column volumes of 16 mM DDM, 250 mM KCl, HSlo was eluted off the column with 100 µM Flag peptide (Sigma) in 16 mM DDM, 250 mM KCl. The eluate was separated on SDS-PAGE using 4–15% ReadyGel (BioRad), and protein detected with Gel-Blue staining solution (Pierce). For western blots, protein was wet transferred to PVDF membrane, blocked with PBS, 5% non-fat milk, 0.05% Tween-20 followed by incubation with the appropriate primary antibody in blocking solution. Blots were washed extensively in PBS, 0.05% Tween-20 before incubation with the appropriate secondary antibody. Secondary antibodies were matched with the corresponding primary antibody. These included bovine anti-goat IgG-HRP (mouse/human cross-adsorbed), donkey anti-rabbit IgG-HRP (mouse/human crossadsorbed), and chicken anti-mouse IgG-HRP (human cross-adsorbed) from Santa Cruz. Blots were again washed extensively in PBS, 0.05% Tween-20 before enhanced chemilluminescence (ECL) detection. WestDureUltra (Pierce) substrates were used for ECL detection.

### siRNAs

In experiments using HEK cells siRNAs were transfected using Oligofectamine or RNAiMax (Invitrogen) at a final concentration of ∼80 nM total siRNAs. Paired plates of cells were transfected with siRNAs or buffer, then either stained live with anti-Flag/488 for surface protein or stained after fixation and permeabilization for total HSlo protein. Assays were done 48–72 hrs after transfection. Unless indicated otherwise, a combination of siRNAs against the same gene were used. siRNAs to HSlo and β-catenin were purchased from Qiagen and IDT. siRNA sequences to β-catenin (sense) are: 5′r(UGCUUGGUUCACCAGUGGAUU)3′and r(GGUGUAGAACACUAAUUAA)d(TT); siRNA sequences to HSlo (sense) are 5′r(GGGAUGUUACGUCAACCAU)D(TT)3′ AND 5′R(GGCGGAUGGCACUCUCAAA)d(TT)3′; siRNA sequences to β4 (sense) are 5′r(UUUCUGCUUGUACGUGACAUU)3′. In these experiments, cells were transfected using 40 nM of each siRNA, or 80 nM for β4.

In experiments on hair cells we used three siRNAs to chick β-catenin that had the following sequence: CTNNB1-1 CAACCAAGCAGGAGGGAAUUU (sense); CTNNB1-2 CAACAAGACAAAUGUGAAAUU (sense); CTNNB1-3 CCAUGGAGCCAGACAGAAAUU (sense). Here siRNA was transfected at a final concentration of 33.3 nm each (for a total concentration of 100 nM). In these experiments we used the x-TREMEGene SiRNA Transfection Reagent (Roche, Mannheim, Germany) as previously described [26]. Institutional Animal Care and Use Committee (IACUC) at Yale University specifically approved this study (protocol number 2010-10439 “Studies on Hair Cell BK Channels”).

### TOPFLASH Luciferase Reporter Assay

HEK 293T cells grown in 12-well culture plates were transiently transfected with TOP-FLASH TCF reporter plasmid (0.2 µg), β-galactosidase expression vector (0.2 µg) and various expression plasmids as indicated for individual experiments. The total quantity of DNA (1.6 µg) added to each well for each of the transfection protocols was held constant by adding mock DNA (pcDNA3.1) where necessary. Cells were maintained in serum replete medium and then harvested 24–30 h post-transfection in 300 µl cell lysis buffer (Promega). Luciferase activity was determined using a luciferase assay system (Promega) and luminometer according to the manufacturer's specifications. β-galactosidase activity was measured at 420 nm using a spectrophotometer and was used to normalize for transfection efficiency. An aliquot from the cell lysates of each well were saved and used to determine protein expression of the transfected plasmids by Western blotting.

### Electrophysiological recording

Macro-patch currents were obtained in the inside-out patch-clamp configuration [27] at room temperature from stable cell lines expressing HSlo and the different HSlo mutants. Patch pipettes (∼1 MΩ) were fabricated from borosilicate glass capillaries (WPI). Because of the high levels of HSlo expression, 21 nM Ibtx was included in the pipette to partially block the patch current *I*. Only records where the estimated maximum series resistance (*R_s_*) voltage error *R_s_I* was less than 10 mV were used for analysis.

The pipette solution contained (in mM): 140 KCl, 20 KOH, 10 Hepes and 2 MgCl_2_ (pH 7.2). The bath solutions contained (in mM): 140 KCl, 20 KOH, 10 Hepes, 5 mM EGTA (for 0 Ca^2+^) or 5 mM HEGTA (for 10 µM Ca^2+^), and added CaCl_2_ to reach 10 µM free Ca^2+^ concentration (pH 7.2). The amount of CaCl_2_ needed to obtain 10 µM Ca^2+^ was calculated using Max Chelator (http://www.stanford.edu/~cpatton/downloads.htm). The desired free Ca^2+^ concentration was confirmed with a Ca^2+^-sensitive electrode (Orion electrode, Thermo Labsystems). Following initial recording in 0 µM Ca^2+^, currents were then recorded in 10 µM Ca^2+^ after fast perfusion exchange using a QMM perfusion tip (ALA Scientific Instruments). Activation time constants were determined by single exponential fitting of the traces following a 150 µs delay.

### HSlo peptide array

HSlo peptide arrays were made by JPT Peptide Technologies (Berlin, Germany). Glass slides were spotted with individual peptides. Human and mouse IgG spots were used as controls and served to normalize fluorescence intensity between arrays. Purified GST-β-catenin in PBS was incubated with each triplicate array at a concentration of 1 µg/ml for 1 hour, followed by 3 washes in PBS. Bound GST- β-catenin was detected by 1∶1000 rabbit anti GST antibody followed by 3 washes in PBS, 0.05% Tween-20 and incubating with Alexa 546 conjugated goat anti-rabbit antibody (1∶5000). The slides were washed ×3 with PBS, 0.05% Tween-20 dried and fluorescence detected on a Genepix 4000B Microarray Scanner (Axon Instruments, CA). Fluorescence intensity was normalized to mouse IgG controls. A second triplicate set of arrays probed with GST alone and processed in an identical manner served to identify non-specific binding. Mean fluorescence data from each spot was averaged between the triplicate arrays. Mean fluorescence intensity for each peptide from arrays probed with GST alone was subtracted from corresponding data from arrays probed with GST- β-catenin to obtain an index of binding of β-catenin to each peptide. Fluorescence intensity was expressed in arbitrary units. We noted a range of values from 0–60,000 A.U. We considered a peptide to have true binding when it along with the two adjacent peptides had fluorescence intensity above 10,000 A.U.

### Competitive HSlo binding to β-catenin

Cell lysates were made as in immuno-precipitation experiments. Slo peptides used for competitive binding were made by Biomatik (Canada). Peptide AchE is a 15mer fragment from acetylcholinesterase, used as a negative control. All peptides were dissolved to make 1 mg/ml stock solution, and used at a final working concentration of 100 µg/ml in peptide competitive binding assays. Briefly, 50 µl glutathione/agarose beads (from Pierce) were incubated with 1.5 µg of purified GST-tagged β-catenin (CTNNB1 from Abnova, Taiwan) in 0.5 ml wash buffer (same as IP wash buffer) for 30 min at 4°C. After washing, 20 µg of each peptide together with 0.2 ml HSlo HEK cell lysate (with fresh protease inhibitors added) was mixed with the β-catenin-bound beads, and incubated at 4°C over night. After extensive washing with wash buffer the bound HSlo- β-catenin was eluted by incubating with 50 µl 4× SDS sample buffer at room temperature for 30 min and collected by centrifugation. Western blotting, chemilluminescence detection, and densitometry measurement protocols were as described as in co-IP experiments.

## Results

### HSlo channels are distributed close to adhesion junctions, and colocalize with β-catenin on the surface of HEK cells

**Figure 1:**
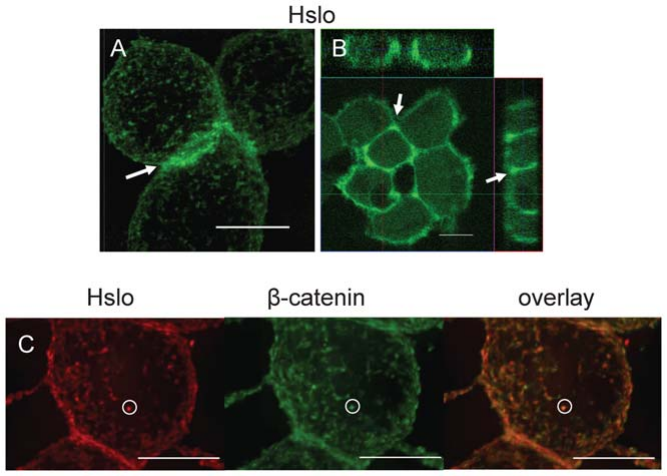
HSlo expression on HEK293 cell surface is concentrated at adhesion junctions and is co-localized with β-catenin. (A) Projected 3-D reconstruction from a Z-series of fluorescence images of HSlo-HEK cells surface-labeled with Ibtx-biotin and streptavidin-Alexa488 conjugates. HSlo has a punctate distribution on the cell surface, and is clearly concentrated at cell adhesion junctions (shown by the arrows). (B) Orthogonal view of confocal images of the surface-labeled HSlo-HEK cells. Note the relative abundance of HSlo near cell-cell contacts. (C) Cells were first surface labeled for HSlo (red) as above, and then permeabilized for labeling with mouse anti- β-catenin antibody (green). Co-localized spots are shown in orange (circle as an example) in the overlay image from 3D reconstructed projection. There is strong co-localization of HSlo and β-catenin at the cell surface. Co-localization analysis using the JACoP plugin in Image J confirm a strong co-localization between the β-catenin and HSlo; Pearson's correlation was 0.748, Li's intensity correlation quotient was 0.38 and the Manders M1 (fraction of catenin overlapping with HSlo) and M2 (fraction of HSlo overlapping with catenin) coefficients were 0.991 and 0.947 respectively. Scale bars are 10 µm.

To study β-catenin-HSlo interactions we used an HSlo-HEK stable cell line, and ascertained the distribution of HSlo on the surface of these cells. We labeled Slo in these cells with a biotin-derivative of the highly specific BK channel blocker Iberiotoxin (Ibtx), which was in turn labeled with streptavidin-Alexa488 conjugate [22]. As shown in both the projected 3D reconstructions (Figure 1A) and orthogonal sections of confocal images (Figure 1B), there was a punctate pattern of surface labeling, concentrated near cell-cell junction in these cells. This pattern of staining was reminiscent of cell adhesion complexes, and suggested that this cell line could serve as a model system to study interactions between HSlo and β-catenin.

We then sought to determine if β-catenin colocalize with HSlo in these cells. Staining for the cell surface population of HSlo channels revealed sharp co-localization of these channels with β-catenin (Figure 1C).

### HSlo S10 deletion mutants have decreased surface expression compared to wt HSlo

**Figure 2:**
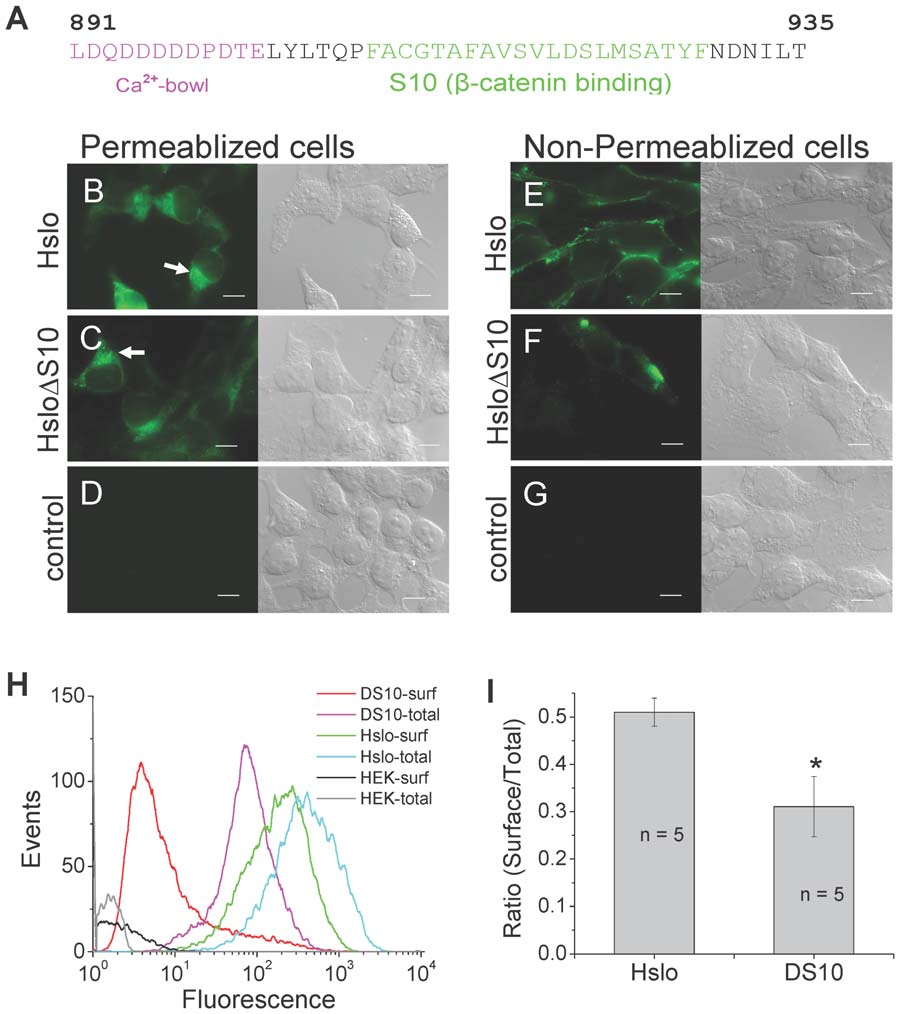
Mutant channel with deletion of the S10 region has decreased surface expression in a stable HEK293 cell line. (A) Sequence of the Slo channel near the S10 region, which follows closely after the Ca^2+^ bowl region. (B, E) Immuno-fluorescence images showing HSlo wt expression detected by anti-FLAG antibody. Permeabilized cells show total expression (B), while non-permeabilized cells (E) show only surface expression. (C, F) mutant with the S10 region deleted (HSloΔS10) has reduced surface expression of HSlo. Here the exposure conditions for this pair of micrographs were chosen to give (part C) approximately the same brightness as part B, to normalize for total HSlo expression. Arrows show similar intracellular distribution of channel proteins in both wt and deletion mutant. Each panel also includes the corresponding DIC image for comparison. (D, G) negative controls using wt-HSlo without primary antibodies. Scale bars = 10 µm. (H, I) FACS quantification of the surface to total ratio of channel expression in wt HSlo and HSloΔS10 mutant (here abbreviated as DS10). Surface to total ratios were obtained by integration of the histograms. There is a 40% decrease in surface protein expression for the HSloΔS10 mutant determined by the surface to total HSlo ratio (+/− SEM, p = 0.032 in two sample t-test .n = 5).

Previous experiments had shown that the S10 region is important for the interaction of Slo with β-catenin [16]. The S10 region, along with the adjacent “calcium bowl,” is highly conserved in Slo channels from *Drosophila* through humans suggesting that this region is critical to the normal functioning of this channel (Figure 2A and Figure S2). To further define the effects of S10, we made a deletion construct lacking the S10 region (HSloΔS10). We established a stable cell line with the construct in HEK cells and examined surface expression of HSlo. As shown in Figure 2, total HSlo decreased although HSlo on the surface of these cells decreased even further. In order to better quantify the changes in total Slo expression, we utilized fluorescence activated cell sorting (FACS). We ascertained surface expression of HSlo by labeling the FLAG tag at its extracellular N-terminus in non-permeabilized cells. Total expression of HSlo was determined by labeling the FLAG epitope after permeabilization. Figures 2H and I show that the relative surface expression of the deletion mutant decreased approximately 40% compared to wt Slo.

It should be noted that while the total expression of Slo in the deletion mutant was reduced, its expression pattern within the cell, was similar to the expression pattern of HSlo wt.

### β-catenin knockdown decreases HSlo surface expression in stable HSlo-HEK cells

**Figure 3:**
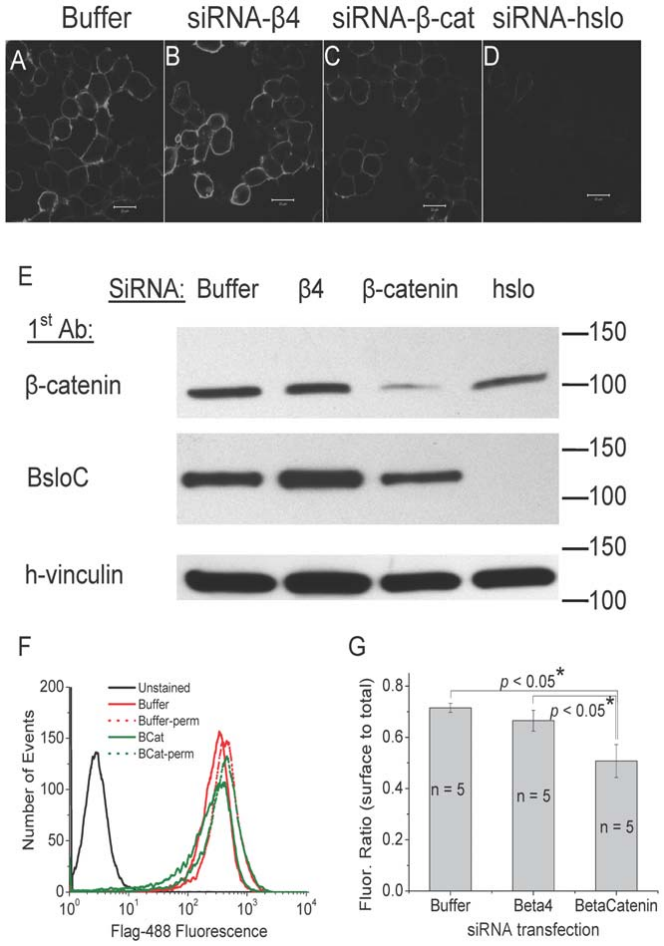
Transfection of siRNA against β-catenin into HSlo-HEK cells decreases surface expression of HSlo. (A–D) HSlo surface expression was detected by anti-FLAG in HSlo- HEK cells transfected with different siRNAs. Compared to cells transfected with either no DNA (A), or with siRNA against the chick β4 subunit of Slo that has no comparable sequence identity in mammals (B), cells transfected with siRNA against β-catenin have decreased surface labeling (C). As expected, transfection of siRNA against HSlo eliminates nearly all surface HSlo signal (D). Scale bars = 20 µm. (E) Western blot analysis showed the efficiency and specificity of HSlo and β-catenin siRNA knockdown of protein expression BsloC, a rabbit polyclonal antibody against the very C-terminus of bovine Slo (BsloC) that shows high sequence homology with hSlo, was used for detection of Slo. Detection of h-vinculin was used to confirm equivalent loading of wells. (F, G). FACS experiments show the decrease in the ratio of surface to total protein expression. This ratio was obtained from the integration of the histograms. Figure 3G shows that relative surface HSlo expression decreases 29% in β-catenin siRNA transfected cells compared to control transfected cells (+/− SEM). One way ANOVA p = 0.0179. In a Student-Newman-Keuls Multiple Comparisons Test between 1. siRNA beta catenin and siRNA chick beta-4 p<0.05; 2. mock transfected cells and beta-catenin siRNA treated cells p<0.05; and 3. mock transfected cells and beta-4 siRNA treated cells p>0.05.).

Since the S10 region is important for surface expression and since interaction of HSlo with β-catenin was thought to be mediated by the S10 region, we hypothesized that β-catenin was important for the surface expression of Slo through its interaction with Slo at the S10 region. We tested this hypothesis by knocking down the expression of β-catenin using siRNA, and then determining surface expression of HSlo in HEK cells. As shown in Figure 3, knocking down β-catenin with siRNA resulted in a decrease in the surface expression of HSlo (Figure 3C). As a control we used siRNA against the beta-4 subunit of chicken Slo (cSlo) which is not expressed in these cells. We noted no decrease in the surface expression of HSlo when transfected with siRNA to the chick β4 of the BK channel whose sequence has no homology in mammals. Also as a control we observed that siRNA to HSlo produced a marked decrease in the surface expression of HSlo. Again, we performed FACS experiments to quantify these effects. The ratio of surface to total HSlo decreased 29% in siRNA-β-catenin transfected cells compared to control transfection (Figure 3G). Interestingly, the transfection with β-catenin siRNA decreased the total amount of HSlo expressed in these cells, as detected by western blots of cell lysates (Figure 3E) and FACS using permeabilized cells. Moreover, we also detected more HSlo fragments in all the HSloΔS10-HEK cell lines compared to the wt HSlo (see Figure S1.). This suggests that interaction with β-catenin may be important in preventing HSlo degradation.

### β-catenin knockdown decreases Slo clusters on the surface of chick hair cells

**Figure 4:**
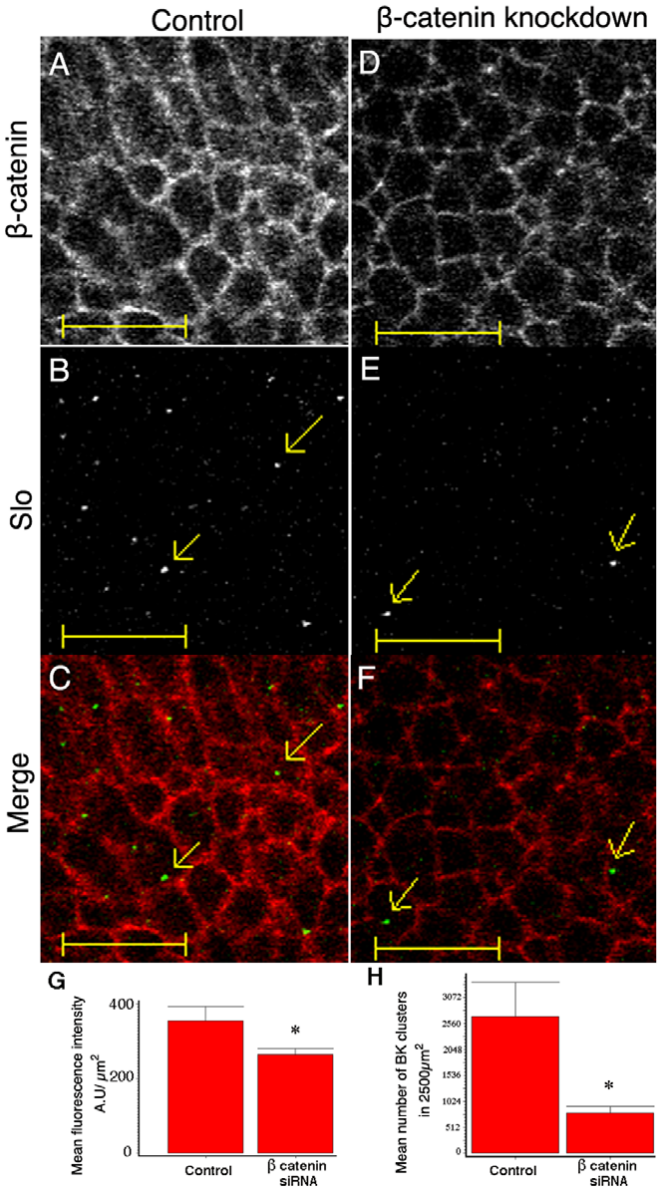
Total Slo expression and more significantly, surface clusters of Slo on the surface of hair cells are decreased with β-catenin knockdown. Shown are confocal images in the X-Y plane of control tall hair cells (A, B and C) and hair cells transfected with siRNA to β-catenin (D, E, and F) stained with antibodies to β-catenin (A,D) and Slo (B,E). The corresponding pseudo colored (β-catenin in red and Slo in green) merged images are shown in C and F. Slo clusters of varying intensities on the surface of the cell are indicated (yellow arrows). Note that the thresholds for the Slo images have been changed to show Slo clusters only. These images were obtained at approximately the same z plane depth. There is a 20% reduction in β-catenin staining after knockdown (mean fluorescence intensity 812+/−31 S.E.M. control vs. 646+/−30 S.E.M., p = 0.003 on a t test, n = 3). There is a reduction in Slo staining (G) and Slo clusters (E,H) after β-catenin knockdown. Mean fluorescence intensity of Slo per µm^2^ in control hair cells in a 2500 µm^2^ area was 366 A.U. (+/−21 S.E.M, n = 3 cochlea) vs. 266 A.U. (+/−8) in β-catenin siRNA treated hair cells (p = 0.018 on a t test, n = 3 cochlea). There is an even greater reduction (70%) in Slo clusters after β-catenin knockdown (E) in a similar 50 µ×50 µ area. The number of Slo clusters in control tall hair cells were 2695 (+/−675 S.E.M., n = 3 cochlea) and contrasts with the 790 (+/−132 S.E.M., n = 3) Slo clusters in tall hair cells from β-catenin siRNA treated cochlea (p = 0.025 on a t test). Also note that we were unable to quantify co-localization between Slo and β-catenin in hair cells, since Slo forms clusters in these cells, unlike β-catenin, which has a more uniform distribution. In contrast, in HEK cells both proteins have a more uniform distribution with wide overlap. Manders overlap co-efficient (M1) in hair cells was 0.015, effectively ruling out meaningful co-localization analysis.

The initial rationale that led to the detection of interactions between Slo and β-catenin was to ascertain mechanisms that led to Slo localization at the basolateral surface of hair cells (along with voltage gated Ca^2+^ channels). We therefore sought to determine how β-catenin knockdown would affect Slo expression in hair cells. In these experiments we used chick hair cells in culture and knocked down β-catenin using siRNA transfection. As shown in Figure 4 there was a significant reduction in Slo expression in tall hair cells that receive afferent innervation. Total Slo expression was reduced 25% while β-catenin showed a 20% reduction. More importantly we noted a greater (70%) and statistically significant reduction of Slo clusters on the surface of hair cells after β-catenin knockdown. We have previously established that Slo channels exist as clusters on the basolateral surface of hair cell membranes [24]. Since almost all the Slo on the membrane of these cells are present in clusters, and since we did not observe Slo on the membrane independent of these clusters in siRNA treated cells, we cannot differentiate whether the effects on Slo clustering were due to decreased membrane targeting or effects on channel clustering.

### In H28 β-catenin null cells, surface HSlo expression is stimulated by high-level exogenous β-catenin co-expression

**Figure 5:**
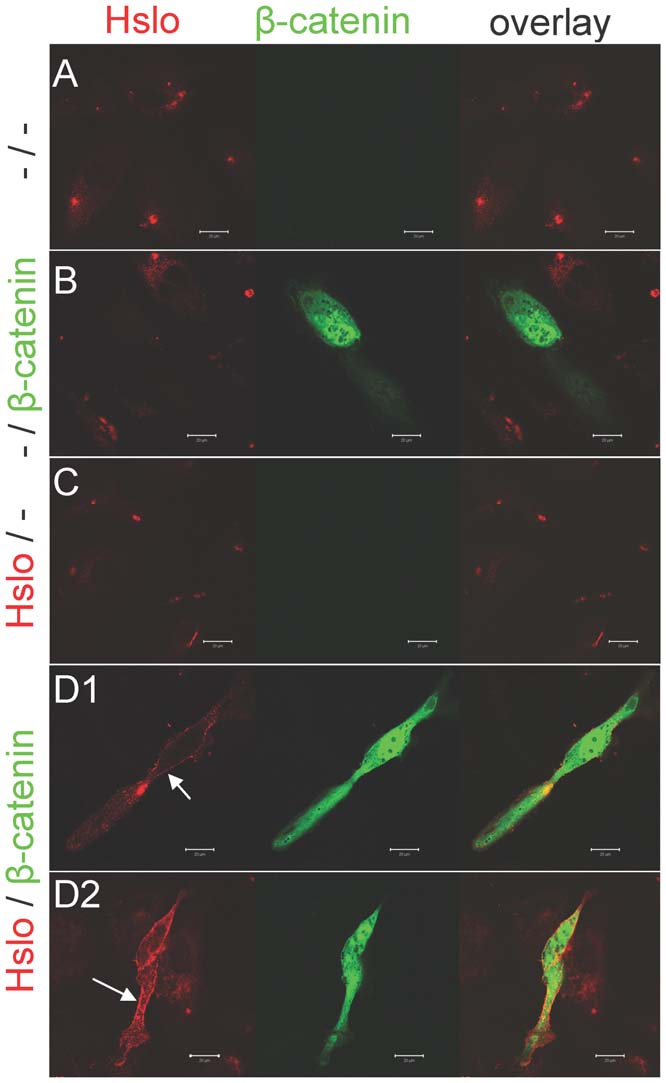
HSlo surface expression in β-catenin -null H28 cells occurs only with co-expression of β-catenin. HSlo was surface-labeled with anti-Flag antibody and Alexa594-conjugated 2nd antibody (red), while β-catenin was N-terminally tagged with EGFP (green). HSlo surface expression was not detected when cells were transfected with empty vector (A), or EGFP- β-catenin alone (B). There was minimal surface expression of Slo with transfection with HSlo alone (C). However, when H28 cells were co-transfected with both HSlo and EGFP- β-catenin, surface expression of HSlo can be seen (arrows) in the cells having a high level of β-catenin expression (D1, D2). All scale bars = 20 µm.

We also tested the expression of HSlo in the β-catenin-deficient mesothelioma cell line H28 [28]. H28 contains a homozygous deletion of the β-catenin gene, making it useful in studying cellular processes involving β-catenin [28] [29]. Since these cells have no endogenous β-catenin expression, we reasoned that the surface expression of HSlo would be hindered. When H28 cells were transfected with the HSlo construct, very little surface expression of HSlo was observed (Figure 5C). However, when H28 cells were co-transfected with HSlo and an EGFP-tagged β-catenin, high HSlo surface expression was observed only in cells with high-level β-catenin expression (Figure 5D). Thus the surface expression of HSlo is correlated with the amount of β-catenin expressed.

### β-catenin co-immunoprecipitates with HSlo

**Figure 6:**
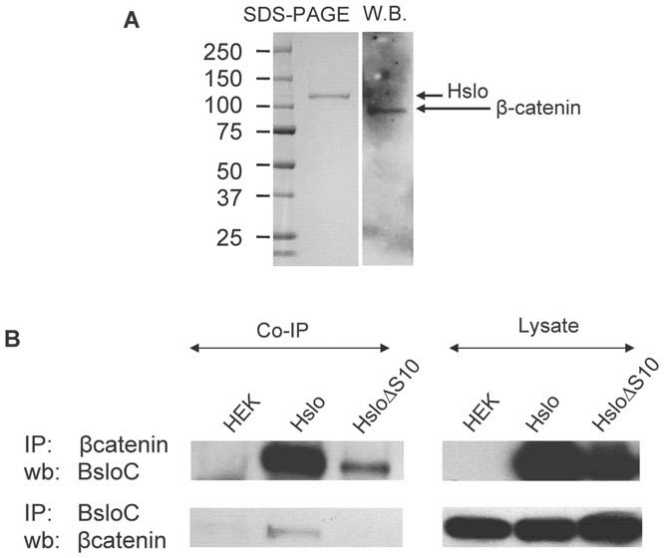
β-catenin co-immunoprecipitates with HSlo from HSlo-HEK cells. (A) β-catenin co-purifies with HSlo. N-terminal FLAG-tagged HSlo was purified from HSlo-HEK cells using an anti-FLAG agarose column. Protein eluted with FLAG peptide was separated by SDS-PAGE. Coomassie staining revealed a single band that corresponds in size to HSlo (120 kDa). A western blot from the same gel, probed with mouse anti β-catenin antibody revealed a single band that corresponds in size to β-catenin (∼90 kDa). (B) Reciprocal immunoprecipitation experiments using protein A/G agarose and a rabbit antibody against the very C-terminus of bovine Slo (BsloC) revealed that HSlo was present in β-catenin immunoprecipitates (upper panel) and vice versa (lower panel). On the other hand, the S10 deletion mutant HSloΔS10 has weaker Slo signal than the wild type HSlo in β-catenin immunoprecipitates. The ratio of HSlo in immunoprecipitates from wild type HSlo∶ HSloΔS10 was 4∶1. The ratio of HSlo from lysates of WT-HSlo and HSloΔS10 was 2∶1. Thus even when corrected for reduced expression of HSlo in the HSloΔS10 cell line, there was a reduction in the amount of HSlo immunoprecipitated by β-catenin. When immunoprecipitated with BsloC, β-catenin was not detectable in HSloΔS10 pulldown, indicating a weaker interaction with β-catenin.

The data presented up to this point clearly show the interaction between β-catenin and HSlo in the S10 region of HSlo is important for channel surface expression, and possibly also affecting channel stability. Lesage at al were unable to show a direct interaction between these proteins by immunoprecipitation in COS cells (although they were able to do so in whole brains) leading them to conclude that this interaction was indirect. We tested the binding between β-catenin and HSlo by purifying HSlo tagged with the FLAG epitope. We column-purified HSlo from HEK cell lysates using an anti-FLAG antibody. β-catenin was detected by western blotting in purified HSlo sample (Figure 6A). Conversely, we were able to detect a robust signal for HSlo in western blots of immunoprecipitates of β-catenin (Figure 6B). Together, these results are consistent with a stable interaction between β -catenin and HSlo.

We also tested for co-immunoprecipitation with the deletion mutant HSloΔS10. β-catenin immunoprecipitates from stable cells expressing the deletion construct showed reduced levels of HSlo (Figure 6B). Consistent with this observation we were unable to detect β-catenin in immunoprecipitates of HSlo in cells expressing mutant HSloΔS10. This would argue for a stable interaction between β-catenin and HSlo that is partially dependent on the participation of amino acids in the S10 region of HSlo.

### Phosphorylation mutants in the S10 region affect expression patterns and channel kinetics

Our co-IP data indicates that the mechanism of the interaction of HSlo with β-catenin through the S10 region and its significance in HSlo surface expression may lie in a simple binding between these two proteins. However, other factors may modulate this interaction or its physiological consequences. For instance, there are two potential GSK phosphorylation sites in the S10 region that contain the consensus GSK phosphorylation motif (SXXXS). A recent work attempting to identify putative phosphorylation sites in Slo determined these sites to have a high probability of phosphorylation based on two phospho-prediction algorithms [30]. These sites were however not accessible to liquid chromatography tandem mass spectrometry analysis [30]. Since they are in the S10 region, we sought to determine if these putative phosphorylation sites affect the surface expression of HSlo. To test this possibility we substituted the two serine residues S918 and S922 with alanine (S10AA) to mimic a constitutively dephosphorylated state. A second construct was made where these two serine residues were substituted with aspartate (S10DD) to mimic a constitutively phosphorylated state. Monoclonal stable cell lines made with these two respective constructs that have good HSlo expression, as assayed by western blotting, were chosen for further study.

**Figure 7:**
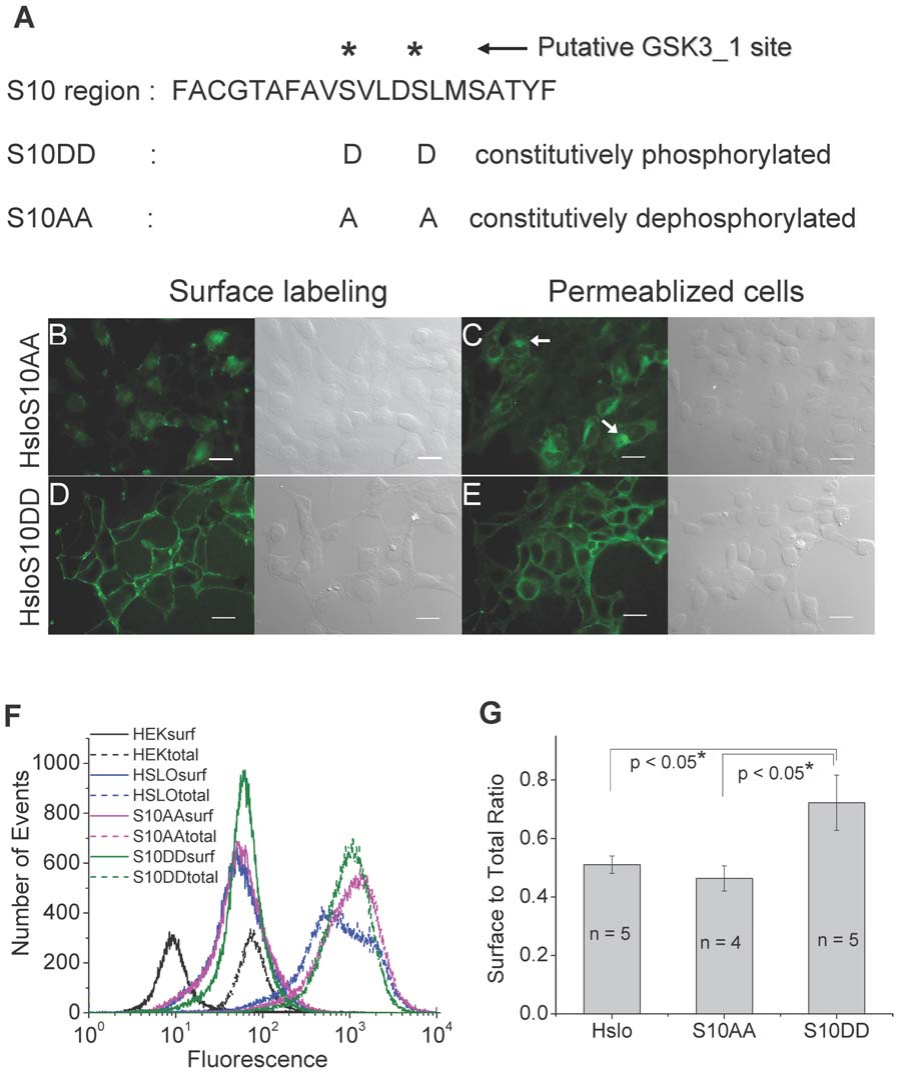
Mutation of the putative GSK3_1 phosphorylation sites in the S10 region has clear effects on HSlo surface expression. (A) Construct for the mutants at the GSK3_1 site (SXXXS) on S10. The S10AA phosphonull mutant has very weak surface expression (B) with prominent intracellular ER-like retention (arrows in C), while the S10DD phosphomimetic mutant has excellent cell surface expression (D) with minimal ER-like retention (E). Since the total expression of the S10AA mutant was lower than that of S10DD according to western blotting results, we acquired panels (B) and (C) with identical exposure times that were longer than those for panels (D) and (E), so that the fluorescence intensity in the images of permeabilized cells C and E is comparable. Scale bars = 20 µm. FACS experiments gave similar results (F). Each sample represents the fluorescence distribution of 20,000 cells. (G) Further quantification of the ratio of surface to total protein expression using FACS histogram integration on sample sets with surface labeling and total labeling (+/− SEM). A one-way ANOVA yielded a p value of 0.036. A multiple comparison test revealed significant differences between HSlo and S10DD (p<0.05), and S10AA and S10DD (p<0.05).

The surface expression of the S10AA mutant is markedly reduced compared with the S10DD construct, which shows an increased level of surface expression of HSlo (Figure 7). In permeabilized cells, both had similar amount of intracellular labeling of HSlo, although the intracellular distribution of Slo differed. The internal Slo expression is less in S10DD mutant, while S10AA mutant has more intracellular retention (arrows in Figure 7C). We quantified the surface labeling in FACS experiments. Surface expression of the S10AA mutant was lower than wt HSlo. In contrast, the S10DD mutant had the highest amounts of HSlo relative surface expression as determined by the ratio of surface to total labeled HSlo.

**Figure 8:**
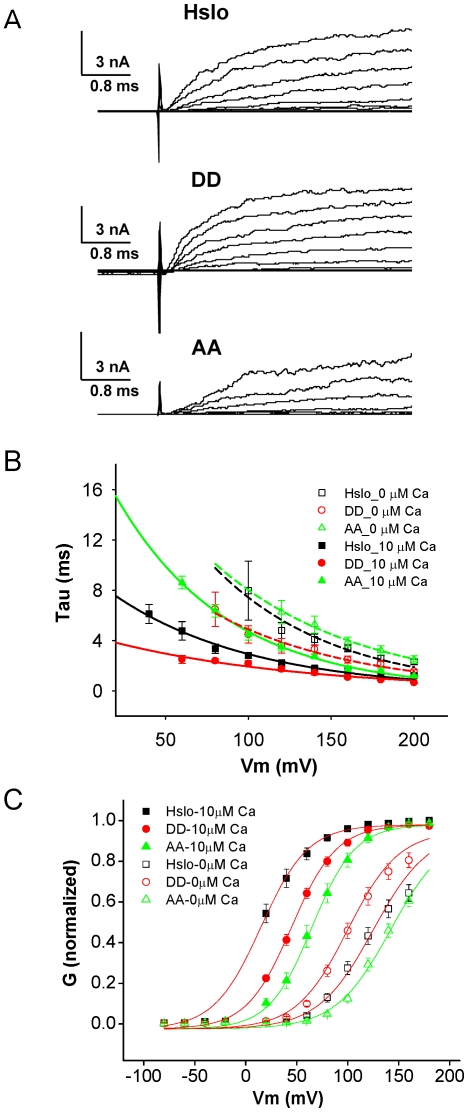
Phosphorylation-mutation effects on HSlo kinetics. (A) Representative current traces from inside-out patch recordings of the wild type and mutant HSlo channels in nominally 0 µM Ca^2+^ demonstrating faster activation of S10DD channels and slower activation of S10AA channels compared to the wild type HSlo. (B) Corresponding activation time constants, obtained from monoexponential fits to the activation time course at the potentials given. Error bars represent SEM from 11–16 patches. The purpose of fitting the time course with a single exponential decay is to make it easier to distinguish the groups between each other. Dashed lines are for 0 µM Ca^2+^, solid lines for 10 µM Ca^2+^, (C) G-V curves. Solid symbols are with 10 µM internal Ca^2+^, while open symbols are with zero Ca^2+^. The V_1/2_ of activation in 10 µM Ca2+ was 20 mV for HSlo, 46 mV for S10DD (DD), and 67 mV for S10AA (AA). In zero Ca2+ the wild type channel had V_1/2_ = 133 mV, while the S10DD and S10AA mutants were 107 and 146 mV.

We also analyzed the effects of the phosphomutants on HSlo channel activity, using macropatch recordings in the inside-out configuration. Two effects were evident. First, these mutations have an effect on the activation kinetics of the channel. In response to depolarizing steps the phosphomimetic form of the channel S10DD consistently activates more rapidly than the wildtype HSlo (Fig. 8A and 8B), irrespective of the concentration of internal Ca^2+^ (0 and 10 µM). In contrast, the phosphodeletion form of HSlo (S10AA) activated more slowly (Fig. 8A and 8B).

These mutations also have an effect on the steady-state Ca^2+^ and voltage sensitivities of the channel (Fig. 8C). At nominally zero Ca^2+^, the G-V curve of the phospho-mimetic form S10DD was left-shifted compared to wt Slo, while the phospho-deletion mimetic was right-shifted, requiring larger depolarization for channel opening. However, with 10 µM internal Ca^2+^ both mutant channels showed right-shifted G-V curves.

### HSlo expression inhibits Wnt signaling in TOP Flash assays

Our data show that the interaction between β-catenin and Hslo affects channel surface expression, and that mutations of putative GSK phosphorylation sites in the S10 interaction domain in HSlo change both surface expression and channel kinetics. Since both β-catenin and GSK are involved in the canonical Wnt pathway [31], we speculate that the interaction between β-catenin and HSlo may also affect Wnt signaling. We therefore tested whether HSlo expression could influence β-catenin signaling through the Wnt pathway.

For these experiments we used the TOP-Flash assay to quantify Wnt signaling [32] We used transfection with either a β-catenin plasmid or with the Wnt 3a plasmid in Wnt 3a-sensitive 293T cells to induce Wnt signaling. In these cells a TCF-LEF promoter controls the expression of the downstream luciferase gene. Exogenous β-catenin, or free cytosolic β-catenin stimulated by expression of exogenous Wnt3a ligand, binds to TCF/LEF and activates luciferase transcription.

**Figure 9:**
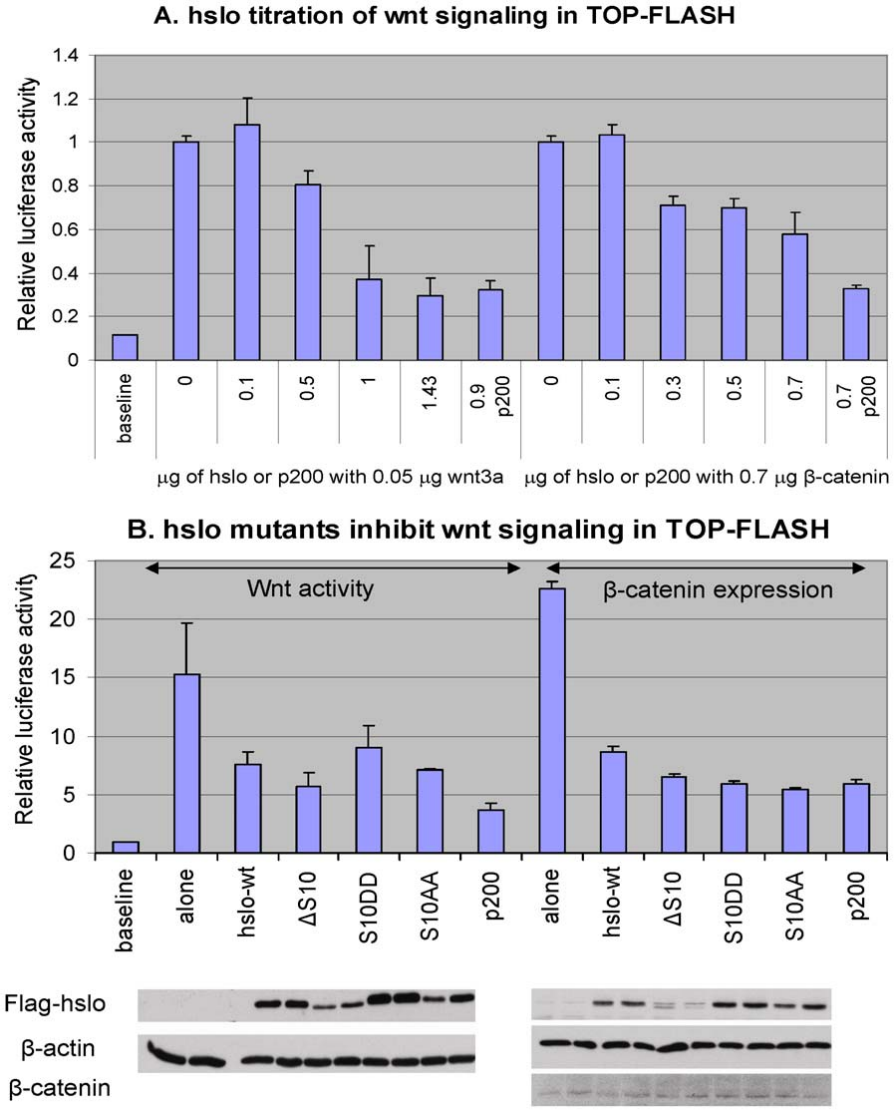
HSlo expression inhibits Wnt signaling in 293T cells as assayed with the TOP-FLASH assay. This assay measures the activity of a luciferase reporter whose expression is under the control of the TCF/LEF promoter to provide an assessment of β-catenin or Wnt ligand-stimulated activation of TCF/LEF mediated transcription. (A) Dose dependent titration of Wnt signaling intensity by increasing amounts of HSlo co-transfection. The normalized relative Wnt signaling intensity decreases when HSlo was co-expressed with either Wnt3a ligand or exogenous β-catenin. The amount of either Wnt3a DNA (on the left side in the bar graph) or β-catenin DNA (on the right side) was fixed and predetermined to give maximum TOP-FLASH responses when HSlo was absent. p200 was used as a positive control. p200 is part of the C-terminus of polycystin-1 protein, and is known to specifically inhibit the binding between TCF-LEF and β- catenin, thus inhibiting luciferase signal. (B) Transfection of HSlo S10 deletion mutant or phosphorylation mutants inhibits Wnt signaling in TOP-FLASH assays in a manner similar to wt HSlo. The data represent means from 3 samples each (+/− SD, n = 3). A total amount of 1.6 µg DNA was transfected into each well with a fixed ratio of HSlo/ HSlo-mutants and Wnt3a or HSlo/ HSlo-mutants and β-catenin. In the Wnt3a experiments we used 1.55 µg of HSlo or HSlo mutants with 0.05 µg of Wnt3a plasmid DNA. In the β-catenin experiments we used 0.9 µg of HSlo or HSlo mutant plasmid DNA and 0.7 µg of β-catenin plasmid DNA.

After establishing the amount of each plasmid needed to produce a sensitive assay, we settled on 0.05 µg Wnt3a or 0.7 µg β-catenin plasmid DNA per well in a 12 well plate. Wnt signaling intensity was assessed by measuring the activity of luciferase [33] [34]. Cells were then co-transfected with increasing concentrations of HSlo plasmid. As shown in Figure 9A, HSlo expression inhibited both Wnt and β-catenin driven signaling in a similar dose-dependent manner. The inhibitory effect on Wnt-signaling was monitored by using p200 as a positive control. p200 is part of the C-terminus of the polycystin-1 protein, and is known to specifically inhibit the binding between TCF-LEF and β-catenin, thus inhibiting the luciferase signal in the TOP-Flash assay [35]. In both assays, about 1.0 µg Slo plasmid per well produces 50% inhibition of signaling. This indicates that while β-catenin affects HSlo surface expression, HSlo expression can also modulate Wnt signaling.

We also tested the effects of various HSlo mutants on Wnt signaling (Figure 9B). Surprisingly, all these mutants have inhibitory effects similar to that of the wild type, even though their channel expression varies significantly (see Figures 2, 7).

### HSlo - β-catenin interaction involves multiple sites on HSlo

**Figure 10:**
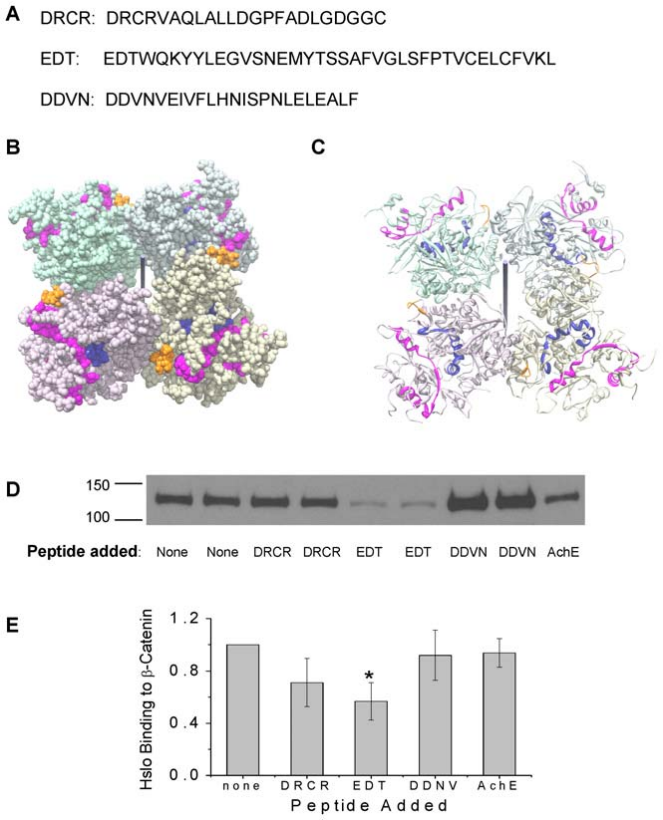
HSlo interaction with β-catenin involves multiple sites on HSlo, including regions outside S10. (A) Three HSlo peptides were chosen for further study from several peptide segments with high binding scores. Shown are the sequences of these peptides. (B, C) Mapping peptide regions to the crystal structure of HSlo C-terminus [36]. The 3D filled –in structure of the C-terminus of Slo (B) viewed at a tangent down the four-fold axis. The black vertical line indicates the four-fold axis. Shown in pink is the peptide sequence beginning at residue E562 (named EDT here), in blue the S10 region and in orange the Ca^2+^ bowl. As is evident the S10 region and the peptide beginning at E562 are adjacent in the tertiary structure of the protein (although separated in the primary structure). C shows the identical view with the ribbon diagram of its secondary structure. As is evident these two regions are on the surface of the protein where they are accessible for protein-protein interactions with β-catenin. (D) Western blots of eluted HSlo protein from an *in vitro* competitive binding assay, showing reduced binding of whole HSlo molecule to immobilized β-catenin in the presence of HSlo peptides. Eluate from β-catenin immobilized to columns incubated with cell lysates of HEK cells expressing HSlo in the presence of each peptide was separated on SDS-PAGE in duplicate. A charged peptide fragment from the extracellular protein acetylcholinesterase (AchE) served as a negative control (single lane on the right). (E) Averaged densitometric analysis of competitive binding measurements (+/− SEM, n = 3). In the presence of the peptide beginning at residue E562 HSlo binding to immobilized β-catenin is reduced 43% (*p* = 0.039, one way ANOVA).

The complex behavior of HSlo mutants in TOP-FLASH assays indicates a complexity in HSlo interaction with β-catenin. The fact that the S10 deletion mutant and two phosphorylation mutants behave similarly to wild type HSlo in inhibiting Wnt signaling points to the possibility of multiple modes of interaction between HSlo and β-catenin. One possible explanation for the seemingly incongruent data is that HSlo interacts with β-catenin in regions outside the S10 region. Therefore we set out to ascertain regions of interaction in HSlo with beta catenin using a peptide array. Overlapping 15-mer peptides of the entire C-terminus of HSlo starting from the linker region with the amino acid sequence VFFILG. were imprinted on an array and probed with purified, GST tagged β-catenin. Out of the total of 101 peptides used, twelve segments of the HSlo C-terminus, including the S10 region, were found to have high binding scores (Figures S2 and S3). Each of these segments contains surface-exposed residues according to the crystal structure of the C-terminal segment of Slo. [36]. Figure 10B shows the location of the S10 region and one of these segments beginning at E562, in the structure of the HSlo C-terminus. The location of these peptides on the surface of the protein is consistent with their ability to interact with other proteins.

In order to further validate the importance of these peptides in interactions with β-catenin we chose three peptide segments for further study (Figure 10A). The first 23 amino acid peptide beginning at residue D1014 (DRCR…DGGC) lies after the S10 region, near the caveolin binding site. The second 38 amino acid peptide beginning at residue E562 (EDT…LCFV) lies near the αH and βG region of RCK1 that have been shown to give rise to intracellular charibdotoxin sensitivity. A third 23 amino acid peptide beginning at residue D410 (DDVN…EALF) lies near the beginning of RCK1 region. We also used a hydrophilic 25 amino acid peptide from the extracellular protein acetycholinesterase as a negative control (AchE). This peptide has a high fraction of charged amino acids (10/23) and was used as a control since interactions with the armadillo repeats in β-catenin are thought to be mediated by charged residues – the armadillo repeats were shown to be the regions on β-catenin that interacted with HSlo. These four peptides were used in an *in vitro* competitive binding assay to test the efficacy of HSlo binding to β-catenin. Immobilized β-catenin was incubated with whole cell lysates of HSlo expressing HEK cells in the presence of these peptides. As shown in Figure 10B and 10C, the peptide beginning at residue E562 has significant inhibitory effects on HSlo binding to β-catenin. This confirms that there are sites on HSlo, outside the S10 region, that can interact with β-catenin. Moreover, since we used highly purified β-catenin and peptide arrays in these experiments, these data strongly suggest that the interaction between the two proteins to be direct.

## Discussion

Several lines of evidence show that β-catenin plays an important role in the surface expression of Slo channels. Exogenously expressed Slo channels are co-localized with β-catenin in HEK cells, and deletion of the Slo S10 region, previously reported to bind to β-catenin, result in decreased surface expression of Slo. Moreover, knocking down β-catenin in HEK cells results in reduced HSlo surface expression. Consistent with these data and confirming a physiological consequence to the interaction, we show that knockdown of β-catenin results in a substantial reduction in Slo clusters on the surface of hair cells in the auditory epithelium of the chick. Furthermore, in the H28 cell line lacking β-catenin, the co-transfection of HSlo and β-catenin together results in greatly increased surface expression of HSlo. The interaction with HSlo represents the first instance in which β-catenin has been shown to increase the surface expression of a protein uninvolved in cell adhesion.

Alongside the effects on surface expression of Slo channel, we also have evidence that the interaction between β-catenin and Slo may stabilize the Slo channel and protect it from proteolytic degradation. For instance, transfection with β-catenin siRNA decreases the total amount of HSlo expressed in HSlo HEK cells, as detected by western blots of cell lysates. In chick cochlea treated with β-catenin siRNA, there was a decrease in total Slo expression. Moreover, in screening S10 deletion mutants, we detected significantly more HSlo fragments in all the HSloΔS10-HEK cell lines compared to the wt HSlo under the same conditions (Figure S1). This may be due to loss of protection conferred by β-catenin binding to HSlo, causing the mutant channel protein become less stable and more prone to proteolytic degradation. Alternatively it is possible that other structural changes in this HSlo mutant causes increased exposure to proteases. Further experiments are needed to clarify this.

Does β-catenin exert its effects through a direct interaction with the HSlo potassium channel subunit? Lesage et al. [16] showed that β-catenin can be co-purified with the chicken Slo protein from whole brain lysates, although not from heterologous expression systems. The latter result led these authors to speculate that the interaction between the two proteins was in fact indirect. However, our data suggest that the interaction between the two proteins is direct. We were able to demonstrate direct binding of highly purified β-catenin (99%) and peptide fragments of HSlo. In this context, the failure by Lesage et al. to demonstrate interactions between these proteins using an immunoprecipitation assay in COS cells might be related to the detergents used. They used 1% Triton-X 100 to solubilize cell membranes while we used 0.5% n-dodecyl β-D-maltoside (DDM) in our successful reciprocal immunoprecipitations.

In yeast two-hybrid experiments [16] it was shown that the interaction between Slo and β-catenin was limited to the S10 region. We find that deletion of the S10 region reduces the cell-surface expression of HSlo. Consistent with this result, reverse coimmunoprecipitation experiments show reduced association of the S10 deletion mutant with β-catenin. This supports the notion that reduction in surface expression of the deletion mutants arises partially from decreased β-catenin binding. Since the association was not completely lost with HSloΔS10 in co-IP experiments, the possibility is raised that HSlo interactions with β-catenin may extend to regions outside the S10 region. It is known that yeast two-hybrid experiments can fail to indentify regions of interaction between proteins. Indeed, our HSlo peptide array experiments identified 10 more sites on HSlo that potentially binds to β-catenin. Furthermore, our in vitro competitive binding experiments confirm that at least one of the possible sites outside S10, namely the 38 residue sequence beginning at residue E562, significantly reduces HSlo binding to immobilized β-catenin.

We identified two potential GSK phosphorylation sites within the S10 region. In addition to changing surface expression, phosphomimetic mutations at these sites affect channel kinetics and steady state voltage activation. At nominally 0 µM Ca^2+^, phosphomimetic and phosphodeletional mutations produce a hyperpolarizing and depolarizing shift in G-V curves respectively. However, at 10 µM Ca^2+^, the steady state voltage activation of both mutants is right-shifted compared to the wt channel. It is unclear how these effects might arise, but they are interesting in view of the fact that in the primary sequence the S10 region is adjacent to the Ca^2+^ binding site.

While our data suggest that phosphorylation of the two GSK3 phosphorylation sites in the S10 region have physiological importance, direct evidence of their phosphorylation is lacking. A recent mass spectroscopy analysis of Slo from rat brain categorized the phosphorylation status of these two residues as indeterminate [30]. In this context we have found variable effects on Slo surface expression using GSK3 phosphorylation inhibitors. GSK3 inhibitors variably increase and decrease relative surface expression of Slo. Since GSK3 phosphorylation also decreases β-catenin levels [37], [38], [39], [40], an indirect effect of increasing GSK activity would be predicted to decrease Slo surface expression. Thus, increased GSK3 activity would be predicted to have paradoxical effects on Slo surface expression with direct phosphorylation of Slo increasing Slo surface expression, and indirectly, via phosphorylation of β-catenin, decreasing Slo surface expression. The use of GSK3 inhibitors variably increasing and decreasing Slo surface expression is consistent with the paradoxical direct and indirect effect of GSK3 activity. In the absence of physical evidence of phosphorylation, these data are the best evidence of direct phosphorylation of Slo *in-vivo*.

Similarly, while our data show clear physiological effects of HSlo interactions with β-catenin on the one hand increasing HSlo surface expression and on the other hand decreasing Wnt signaling, we do not have a satisfactory explanation for the seeming discordance between the observed physiological effects of the interaction between these proteins and their physical interaction. Thus, while the phosphomimetic and phosphodeletional forms have differences in surface expression, immunoprecipitations do not show a difference in the strength of their interaction with β-catenin (data not shown). Conversely, we do not observe differences in Wnt signaling between HSlo and the S10 deletional form, even though immunoprecipitations suggest a clear difference in interaction strength. To be sure any number of reasons could explain these seemingly discordant results. These include differences in threshold between the functional assays and immunoprecipitation assays, allosteric effects on β-catenin's transcriptional activity mediated by regions of HSlo outside the S10 region, functional effects between the two proteins additionally mediated by a third protein etc. Irrespective of the exact mechanisms that underlie these phenomena that must await future exploration, these data point to a complex and dynamic interaction between HSlo and β-catenin.

It is widely recognized that β-catenin has a dual cellular function and involved in cell adhesion and, separately, is integral to the developmentally important Wnt signaling pathway. Using a transcriptional assay we find a dose-dependent inhibition of Wnt signaling by HSlo expression. Wnt signaling induced either by expression of the Wnt3a ligand or by overexpression of β-catenin is suppressed as the expression of HSlo is increased. This effect is likely due to HSlo interactions with β-catenin, which may in turn reduce the size of the free pool of β-catenin available for activating Wnt signaling. It is unlikely that HSlo expression led to increased degradation of β-catenin, since the levels of total β-catenin were invariant irrespective of the mutants of HSlo transfected (Fig. 9B).

The observation that HSlo affects Wnt signaling has substantial implications. It is one of the first instances where the expression of an ion channel has been shown to affect signaling possibly independent of its effects on ionic flux. One instance where this has been previously shown is the ability of the *Drosophila* Eag channel to bind and activate Ca^2+^-calmodulin-dependent protein kinase II (CaMKII), independent of calmodulin and autophosphorylation [41]. Similarly, Wnt signaling by β-catenin is also down-regulated by the C-terminus of the transmembrane protein polycystin-1, which associates closely with the TRP channel polycystin-2 [35]. Future experiments will be required to determine whether HSlo plays a physiologically important role as a regulator of Wnt signaling *in vivo*. This is particularly the case since Wnt signaling affects planer cell polarity of hair cells, and since Slo is restricted to the basolateral pole of hair cells.

In conclusion, we extend previous work showing interactions between the S10 region of Slo and β-catenin. In particular we elucidate the physiological significance of this interaction. We show that this interaction is important for surface expression of HSlo, and may be also important for the stability of Slo channel. The binding of these two proteins extends to regions outside the S10 region, as HSlo reversibly precipitates β-catenin in co-IP experiments, while S10 deletion mutant channels have reduced, but not absent, binding to β-catenin. This possibility was confirmed by β-catenin binding to multiple Hslo peptides in a peptide array. Possible phosphorylation by GSK3 on S10 region of HSlo, and the Ca^2+^-binding states of the channel may also affect this interaction, since mutations on the putative phosphorylation sites in the S10 region alters channel activation kinetics and steady state voltage and Ca^2+^ sensitivity. In addition to the effect of β-catenin on Slo channel surface expression, our data show that HSlo overexpression also affects canonical β-catenin-dependent Wnt signaling pathway. The expression of HSlo can, in a dose dependent manner, inhibit Wnt signaling in TOP-Flash assays. This is among the first in a growing literature demonstrating direct effects on Wnt signaling by ion channels.

## Supporting Information

Figure S1 **ΔS10 mutants have more cellular fragments compared with wt HSlo.** Shown is a western blot of several cell lines expressing HSlo (left lane) and HSlo lacking the S10 region (right three lanes, each of which represents one monoclonal cell line each). Cell lysates were separated on SDS-PAGE and western blotted. The blot was probed with an anti-FLAG antibody in turn detected by HRP conjugated anti-mouse antibody using enhanced chemiluminescence (ECL). Extra fragments between 40 kDa to 75 kDa, likely representing breakdown products containing the N-Terminus of the protein, were detected in the three cell lines lacking the S10 region. We interpret these data to suggest that interaction with β-catenin protects HSlo from proteolytic degradation.
(TIFF)

Figure S2 **β-catenin binds to multiple regions of the Slo C-terminus.** Shown is a schematic of the HSlo C-terminus with its recently determined secondary structure superimposed. Alpha helices (blocks) and β-strands (arrows) are indicated beneath the primary sequence. Secondary structure from the RCK1 and RCK2 regions are shown in pink and blue respectively. Twelve contiguous overlapping peptide sequences that bound to β-catenin on peptide arrays are indicated in green. The S10 region that was previously identified as binding to β-catenin is underlined. Note that the actual binding regions are likely to be smaller than indicated since the peptide arrays involved overlapping peptides.
(TIFF)

Figure S3 **Multiple regions of the C-terminus of HSlo binds to β-catenin.** Shown in graph form is fluorescence intensity of β-catenin bound to an HSlo peptide array. Triplicate arrays were incubated with GST- β-catenin and probed with fluorescent-tagged antibodies to GST. GST alone was used to probe a separate set of triplicate arrays and these fluorescence values subtracted from the corresponding values of the arrays probed with GST- β-catenin to obtain a profile of true β-catenin binding. Binding to twelve separate peptide regions that reached an arbitrary cutoff of 10,000 fluorescence units is indicated. The three peptides used for binding correspond to the peaks 2 (D410DNV…), 5 (E562DT..) and 11 (D1014RC…). The S10 region corresponds to the 10^th^ fluorescence peak. While the peptide with the highest binding (peak 5 E562DT) also inhibited interactions between Slo and β-catenin, we are unable to assert a relationship between intensity of fluorescence in the peptide array with strength of protein-protein interactions. In part this is because such an assertion would require the non-trivial assumption that all peptides were synthesized with equal efficacy. The S10 region for instance is resistant to synthesis (and purification) to allow testing in a non-competitive binding assay.
(TIFF)
