# Supplementary figures and images for: Interactions between β-Catenin and the HSlo Potassium Channel Regulates HSlo Surface Expression
Source: PLoS One. 2011 Dec 14;6(12):e28264. doi: 10.1371/journal.pone.0028264 (PMC3237428; doi:10.1371/journal.pone.0028264)

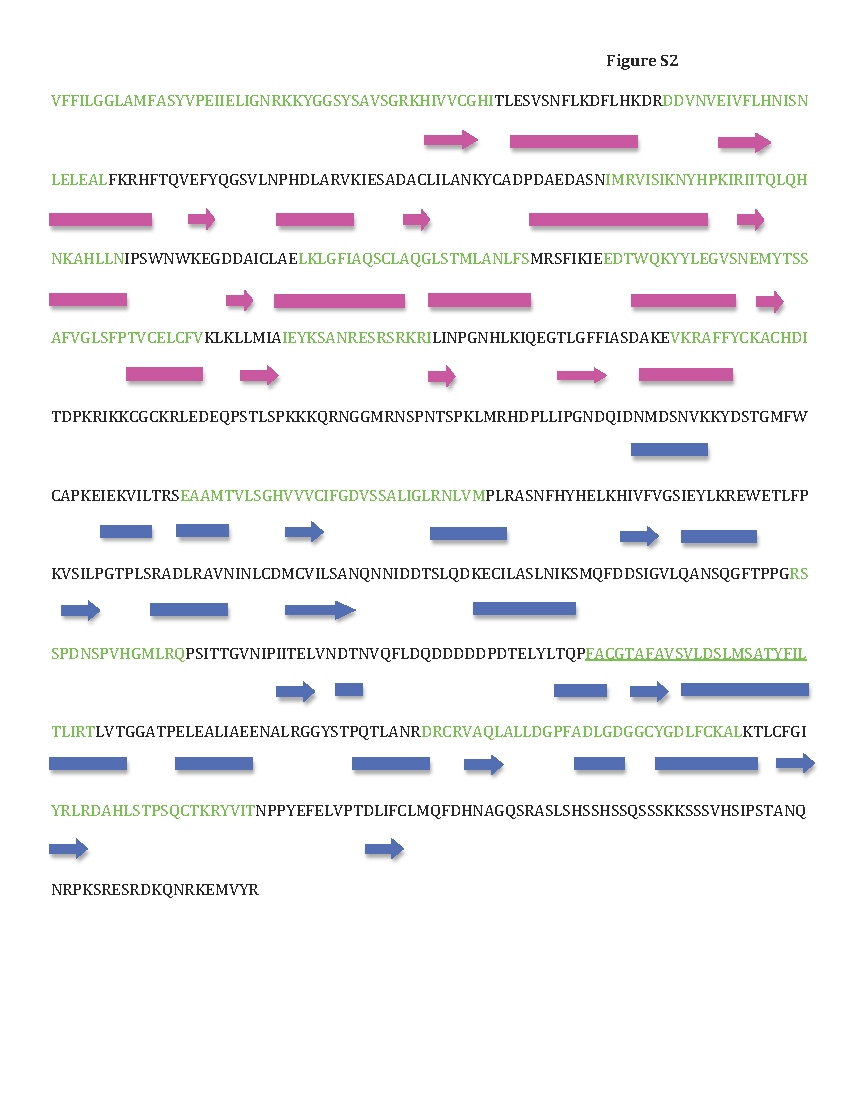

Supplement: Figure S2 — β-catenin binds to multiple regions of the Slo C-terminus. Shown is a schematic of the HSlo C-terminus with its recently determined secondary structure superimposed. Alpha helices (blocks) and β-strands (arrows) are indicated beneath the primary sequence. Secondary structure from the RCK1 and RCK2 regions are shown in pink and blue respectively. Twelve contiguous overlapping peptide sequences that bound to β-catenin on peptide arrays are indicated in green. The S10 region that was previously identified as binding to β-catenin is underlined. Note that the actual binding regions are likely to be smaller than indicated since the peptide arrays involved overlapping peptides. (TIFF) [file pone.0028264.s002.tiff]

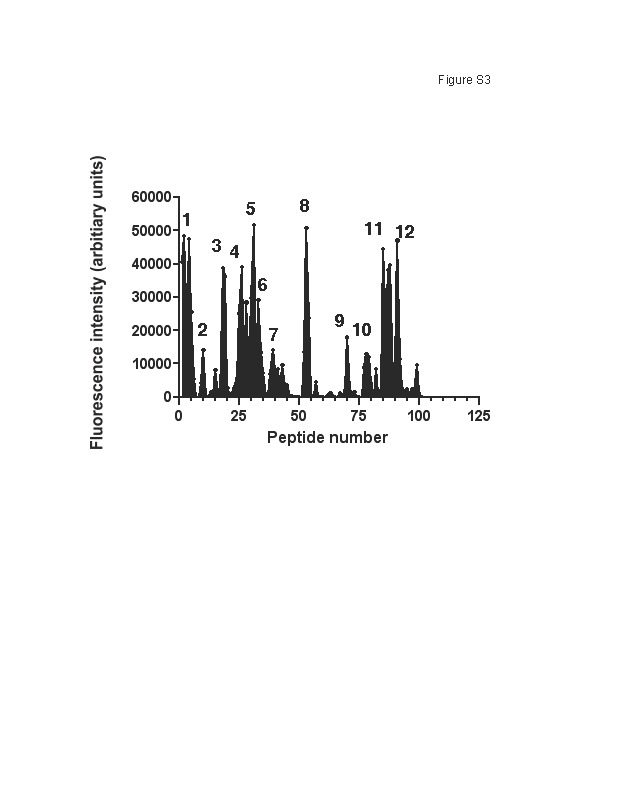

Supplement: Figure S3 — Multiple regions of the C-terminus of HSlo binds to β-catenin. Shown in graph form is fluorescence intensity of β-catenin bound to an HSlo peptide array. Triplicate arrays were incubated with GST- β-catenin and probed with fluorescent-tagged antibodies to GST. GST alone was used to probe a separate set of triplicate arrays and these fluorescence values subtracted from the corresponding values of the arrays probed with GST- β-catenin to obtain a profile of true β-catenin binding. Binding to twelve separate peptide regions that reached an arbitrary cutoff of 10,000 fluorescence units is indicated. The three peptides used for binding correspond to the peaks 2 (D410DNV…), 5 (E562DT..) and 11 (D1014RC…). The S10 region corresponds to the 10th fluorescence peak. While the peptide with the highest binding (peak 5 E562DT) also inhibited interactions between Slo and β-catenin, we are unable to assert a relationship between intensity of fluorescence in the peptide array with strength of protein-protein interactions. In part this is because such an assertion would require the non-trivial assumption that all peptides were synthesized with equal efficacy. The S10 region for instance is resistant to synthesis (and purification) to allow testing in a non-competitive binding assay. (TIFF) [file pone.0028264.s003.tiff]
